# Surgical treatment in Paget’s disease with invasive ductal carcinoma: an observational study based on SEER

**DOI:** 10.1038/srep45510

**Published:** 2017-04-19

**Authors:** Qi Wu, Xiaojun Ding, Juanjuan Li, Si Sun, Shan Zhu, Juan Wu, Qian Liu, Feng Yao, Shengrong Sun

**Affiliations:** 1 Department of Breast and Thyroid Surgery, Renmin Hospital of Wuhan University, Wuhan, Hubei, P. R. China; 2 Department of Clinical Laboratory, Renmin Hospital of Wuhan University, Wuhan, Hubei, P. R. China; 3 Department of Pathology, Renmin Hospital of Wuhan University, Wuhan, Hubei, P. R. China

## Abstract

The aim is to analyse the clinical presentation, treatment and outcomes in patients with Paget’s disease with invasive ductal carcinoma (PD-IDC), with special emphasis on the role of surgical treatment. Using data obtained by the Surveillance, Epidemiology, and End Results (SEER) program from 2010–2013, we investigated the differences in characteristics, overall survival (OS), and breast cancer-specific mortality (BCSM) between patients with PD-IDC and those with invasive ductal carcinoma (IDC). Compared with IDC group, patients with PD-IDC had a better prognosis and lower mortality in adjusted analyses. In the multivariate analysis of cases with PD-IDC, history of ALND was significantly associated with OS while Her2 status were associated with BCSM. Further, subgroup analysis demonstrated no difference between surgical treatment subgroups for either OS or BCSM. The results demonstrated that PD-IDC appears to alter the association between prognosis and Her2 status. Meanwhile, breast-conserving surgery with radiotherapy may be a feasible treatment alternative and sentinel lymph node biopsy should be considered as an appropriate treatment for patients with PD-IDC.

Paget’s disease (PD) of the breast is rare, accounting for 1–3% of all breast malignancies. Paget’s disease is characterized by the eczematous eruption and ulceration of the nipple or areola^1^,^2^,^3^. Due to its rare occurrence, diagnosis of PD may be a delayed or misdiagnosed as benign dermatosis. Breast imaging examination should be used to identify the presence of concomitant breast cancer, and nipple skin biopsy may provide further definitive diagnosis.

In the majority of patients, PD has been found in association with invasive breast cancer or ductal carcinoma *in situ*, with concomitant disease identified in up to 82% to 93%^4^,^5^. Additionally, several studies have found Paget’s disease with invasive ductal carcinoma (PD-IDC) to be associated with tumours that are larger in size and higher in grade as well as negative oestrogen receptor (ER) and progesterone receptor (PR) but positive human epidermal growth factor receptor 2 (Her2) status. Further, diagnosis of PD-IDC may be associated with reduced survival^6^,^7^,^8^,^9^.

Traditional treatment for PD-IDC has been mastectomy due to the common occurrence of sonographically and mammographically hidden multifocal and multicentric malignancies located in breast tissue far from the nipple. However, with the development of imaging technology, breast conservation surgery (BCS) has been found to be a feasible surgical option with low risk for local recurrence in selected patients^5^,^10^,^11^. In addition to BCS, sentinel lymph node biopsy (SLNB) may be effective in patients with PD; however, its role in PD treatment remains unclear. Based on current standards, SLNB has been recommended as a less invasive method than axillary lymph node dissection (ALND) for the staging of patients with early IDC. We speculate SLNB may be effective in detecting ALNs in patients with PD-IDC as well.

For these reasons, the aim of this study was to analyse the clinical presentation, treatment and outcome in patients with PD-IDC, with special emphasis on the role of BCS and SLNB in PD-IDC treatment.

## Results

### Clinical and Tumour Characteristics

A total of 180253 breast cancer patients were eligible during the 2010–2013 study period. We excluded from the analysis 617 patients whose survival times were classified as unknown. A total of 179776 IDC and 477 PD-IDC patients had information available and were included in this study.

Differences in patient demographics, cancer characteristics, treatments, and outcomes between histological subgroups are summarized in Table 1. Compared with IDC, patients with PD-IDC were more likely to have more lymph node involvement and tumours of a higher grade, more advanced stage, and larger size (each P < 0.05). PD-IDC tumours were more likely to be HR-negative and Her2-positive when compared with IDC tumours. Among the treatment options, patients with PD-IDC were more likely to undergo a mastectomy and axillary lymph node dissection but less likely to undergo radiotherapy when compared with IDC patients (P < 0.05).

### Survival Analysis

**Figure 1:**
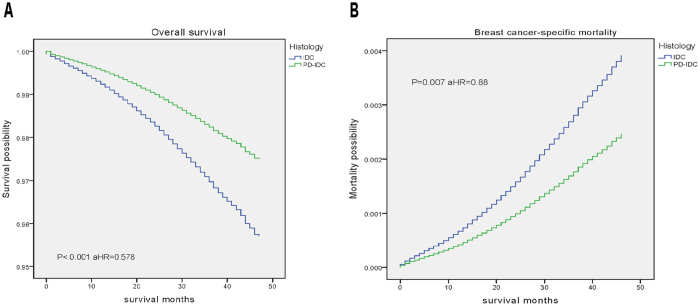
Weighted Kaplan-Meier curves of overall survival (OS) and breast-cancer–specific mortality (BCSM) on histological type. (**A**) OS is based on histological type. (**B**) BCSM is based on histological type. aHR: adjusted hazard ratio (adjusted for age at diagnosis, sex, race, grade, histology, stage, tumor stage, node stage, distant metastasis, laterality, ER, PR, Her2, subtype, radiotherapy, surgery and LN surgery).

Weighted Kaplan-Meier analysis was used to determine OS and BCSM within histological subgroups. At a follow-up time, patients with PD-IDC had an OS of 89.5%, while patient sin the IDC group had an OS of 92.8% (P < 0.05). Further, the BCSM rate was 7.4% within the PD-IDC group compared with 4.5% within the IDC group (P < 0.05) (Table 1). However, patients with PD-IDC had a better prognosis and lower mortality compared with the IDC group in adjusted analyses (Fig. 1). Due to tumour heterogeneity and sample capacity disparity, these may prevent the robustness of the conclusions^12^. Then, we generated 3 independent cohorts by randomly selecting considerable samples from the entirety. Each cohort was required to maintain the same ratio of various factors as that of the original set, without sample overlap among the cohorts. We further analysed the clinicopathologic features and performed survival curves compared each cohort to PD-IDC group (Supplementary 1 and 2). And the results further revealed that patients with PD-IDC had a better prognosis compared with those with IDC.

**Figure 2:**
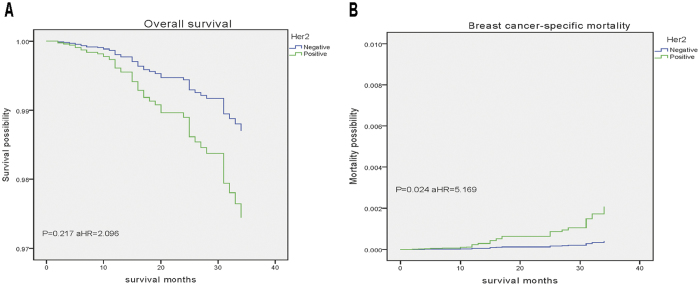
Weighted Kaplan-Meier curves of overall survival (OS) and breast-cancer–specific mortality (BCSM) based on Her2 status in patients with PD-IDC. (**A**) OS is based on Her2 status. (**B**) BCSM is based on Her2 status.

We performed multivariate analysis to evaluate prognostic factors of OS and BCSM in cases with PD-IDC (Table 2). In the multivariate model, tumour stage and history of LN surgery were significantly associated with OS, while age at diagnosis, tumour stage and Her2 status were associated with BCSM (P < 0.05). Among patients, Her2 positive cancer was significantly associated with a higher rate of BCSM, as showed in Fig. 2 (OS, P = 0.217, aHR = 2.096; BCSM, P = 0.024, aHR = 5.169).

### Effect of Surgical Treatment on Survival Outcomes

**Figure 3:**
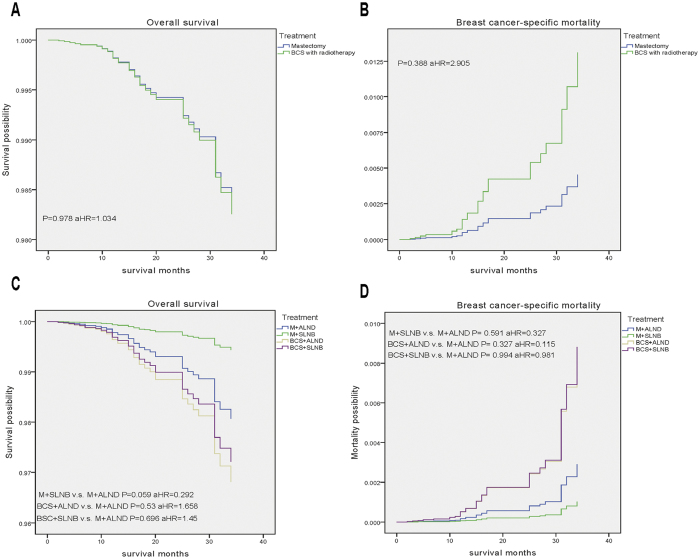
Weighted Kaplan-Meier curves of overall survival (OS) and breast-cancer–specific mortality (BCSM) based on treatment in patients with PD-IDC. OS (**A**) and BCSM (**B**) are illustrated according to type of breast cancer operation. OS (**C**) and BCSM (**D**) are illustrated according to type of axillary operation.

For patients with PD-IDC, the results of multivariate analysis suggested that SLNB was significantly associated with OS during the follow-up period (P = 0.028). Further, we analysed survival outcomes by surgical treatment subgroups. We defined two subgroups based on different surgical treatments: one included patients who underwent BCS with radiotherapy and mastectomy (M), and the other was separated into M + ALND, M + SLNB, BCS + ALND and BCS + SLNB. The results demonstrated no difference between these subgroups for OS or BCSM (Fig. 3).

## Discussion

In this large population-based cohort of cases diagnosed with PD-IDC, we identified improved survival when adjusting for other factors relative to patients with IDC alone. In addition, our analysis of Her2 status demonstrated the PD-IDC patients with Her2 positive cancer were at significantly greater risk of BCSM, as were patients with advanced tumour stage. In our series, there was no difference between surgical treatment subgroups.

In the current study, patients with PD-IDC tended to have a more extensive lymph node involvement and distant metastases and tumours that were higher in grade, more advanced in stage, larger in size, and more frequently HR-positive and that had higher levels of Her2 expression compared to those with IDC. Kothari *et al*.^13^ reported that patients with PD-IDC had a significantly lower survival (10-year OS 49%) than patients with IDC only (64%). They attributed the poorer survival outcomes in patients with PD-IDC to higher levels of Her2-positive expression. Further, they compared the survival of patients with PD to the survival of those with IDC after adjusting for Her2 status and other factors. When controlling for Her2 status, the two groups had a similar OS. However, we analysed common prognostic factors as well as Her2 status. The results showed that patients with PD-IDC had significantly better survival outcomes than those with IDC alone, and patients with Her2-positive cancer had a higher BCSM but similar OS to patients with Her2-negative cancer after adjusting for other prognostic factors. This result differed from Kothari’s study, and this discrepancy may be due to the inclusion of different variables within the two studies. For instance, we included HR status and history of SLNB as variables within our analyses.

Surgical treatment of PD-IDC has been controversial. Historically, patients with PD were predominately treated with mastectomy for two main reasons: the high incidence of potential multifocality and contraindication for BCS patients with centrally located breast cancer. Several studies have revealed that local excision alone was not an appropriate surgical approach for patients with PD of the nipple^3^,^10^. However, the consensus has been that BCS may be effective in selected patients. The previously reported techniques for BCS in patients with PD have varied widely and include nipple excision and central segmentectomy as well as resection plus radiation. There is a place for BCS in selected patients with PD of the breast, especially those with no mass. Long-term follow-up of patients with PD following BCS with radiotherapy has only once been previously reported^14^. This study found that breast cancer-specific survival (BCSS) rate was 91%, 83%, and 76% at 5, 10, and 15 years, respectively. In an analysis using SEER data, Chen *et al*. found a 15-year BCSS up to 61% (95% CI, 53–68%) in patients with PD-IDC diagnosed between 1988 and 2002. Further, there was no statistically difference in survival between central lumpectomy and mastectomy after adjusting for tumour characteristics^6^. Similarly, these studies supported the use of BCS with radiotherapy as a feasible alternative for patients with PD-IDC. Although SLNB may still be considered a controversial treatment in patients with PD, SLNB has become a common approach in patients with breast cancer and appears to be a feasible treatment option^15^,^16^. When IDC has been identified and a mastectomy has been completed, SLNB should still be routinely employed in axillary node negative DCIS patients^17^,^18^. In the present study, patients undergoing SLNB had similar survival to those receiving ALND regardless of surgical mode of their breast cancer treatment. Current National Comprehensive Cancer Network (NCCN) guidelines encouraged axillary staging in patients with PD-IDC, while axillary assessment was not found to be necessary for PD-DCIS undergoing BCS^19^,^20^. Further study regarding the potential benefits of SLNB in patients with PD-IDC is warranted.

A previous examination of the molecular profiles of PD suggest that the luminal B subtype was most frequently identified^21^; however, other studies have reported the HER2-positive (nonluminal) subtype to be more common in primary PD^22^. Some studies have found that tumours in patients with PD were positive for c-erbB-2, cyclin D1, Ki-67 and p16, which have been associated with more aggressive tumour behaviour, and simultaneously had low level expression of Bcl-2 or ER and PR, which has been associated with a better prognosis^21^,^23^. Additional molecular studies are required, and differences in survival outcomes must continue to be monitored.

The main limitations of this study were a small sample size, heterogeneous population and retrospective methodology. The information regarding systemic therapy was insufficient, and follow-up was limited. This may have impacted our results, as Her2-targeted therapy and novel adjuvant radiation may significantly improve survival when fully utilized in the management of PD. Additionally, this study did not include specific information regarding the type of axillary operation and thus used number of lymph nodes excised as a proxy.

Despite these limitations, our study demonstrated that PD-IDC appears to alter the association between prognosis and HER2 status. Meanwhile, BCS with radiotherapy may be a feasible treatment alternative, as it resulted in survival rates similar to those achieved with mastectomy, and SLNB should be considered as an appropriate treatment for patients with PD-IDC. However, surgical treatment plans should be selected based on the results of clinical and imaging assessments. Further studies are needed to minimize variation in treatment of PD-IDC and to establish a standardized management approach for PD-IDC.

## Materials and Methods

### Data source and study design

We obtained data from the National Cancer Institute’s Surveillance, Epidemiology, and End Results (SEER) programme database collected between 2010 and 2013. Her2 status was initially collected by SEER in 2010; therefore, we used 2010 as the starting point for our study. We used the *International Classification of Diseases for Oncology*, 3rd edition (ICD-O-3) histopathology codes to extract data for all cases with Paget’s disease with invasive ductal carcinoma (PD-IDC) (code 8541). Data from patients with ductal carcinoma of no special type (ICD-O-3 code 8500) was obtained to serve as a control group. We selected cases with known hormone receptor (HR) and Her2 status. Patients who did not receive surgery, had ICD of unknown type or were diagnosed at autopsy were excluded.

Type of surgery were categorized as BCS (primary site surgery codes 20–24) of mastectomy (primary site surgery codes 30–80). Because the type of axillary surgery was not reported within SEER, removal of 1–5 lymph nodes was regarded as sentinel lymph node biopsy (SLNB) and removal of >5 lymph nodes removed was regarded as axillary lymph node dissection (ALND), as in previous studies^24^.

Demographic variables included age at diagnosis (<35, 35–49, 50–64, >65 years) and race (white, black, other). Cancer characteristics included stage (I, II, III, IV, unknown), grade (well, moderately, poorly, undifferentiated, unknown), T stage (T0/T1, T2, T3, T4, NA), N stage (N0, N1, N2, N3, NX, NA), distant metastasis (M0, M1, NA), laterality (right, left, paired, bilateral, unknown), and HR and Her2 status (positive, negative, borderline, unknown). Receipt of radiation therapy (no, yes, unknown) was collected to characterize treatment. Tumour subtypes were characterized by a breast subtype variable and defined as HR+/Her2−, HR+/Her2+, HR−/Her2+ and triple-negative (TN).

The two main outcomes in our study were overall survival (OS) and breast cancer-specific mortality (BCSM). Vital status was recorded as “alive” or “dead” in the SEER dataset. Survival time (in months) was calculated for each patient using the “Completed Months of Follow-up” variable in the SEER database. Overall survival (OS) was determined by the proportion of patients alive at the end of the study period or their last follow-up. Breast cancer-specific mortality (BCSM) was determined by the proportion of patients whose cause of death was due to breast cancer relative to that of patients who were alive at the end of the study period or their last follow-up or died due to other causes. Cases without survival times were classified as unknown and removed from the study.

### Statistical analysis

Patient demographics and cancer- and treatment-related characteristics were compared between histological subgroups using chi-square or Fisher’s exact tests. Survival outcomes (OS and BCSM) were estimated using the weighted Kaplan–Meier method, and variables were compared between histological and HER2 status subgroups using log-rank tests. Univariate and multivariate Cox proportional hazard regression models were used to obtain HRs and their respective 95% confidence intervals and estimate relative risk, and these approaches were applied to evaluate the relationship between potential covariates and either OS or BCSM. All statistical analyses were performed and probability of survival curves were generated using SPSS 19.0 (IBM Corporation, Armonk, NY). A two-sided P value < 0.05 was considered statistically significant.

### Ethical approval

This article does not contain any studies with human participants or animals performed by any of the authors.

## Additional Information

**How to cite this article**: Wu, Q. *et al*. Surgical treatment in Paget’s disease with invasive ductal carcinoma: an observational study based on SEER. *Sci. Rep.* **7**, 45510; doi: 10.1038/srep45510 (2017).

**Publisher's note:** Springer Nature remains neutral with regard to jurisdictional claims in published maps and institutional affiliations.

## Supplementary Material

Supplementary Information

## Figures and Tables

**Table 1:** Patient characteristics within subgroups.

Variables	IDC N = 179776(%)	PD-IDC N = 477(%)	P value*
Follow-up(months)	22.09 ± 13.73	22.21 ± 13.62	
Age at diagnosis, y			0.151
<35	3687(2.1)	15(3.1)	
35–49	35024(19.5)	100(21.0)	
50–64	68677(38.2)	164(34.4)	
≥65	72388(40.3)	198(41.5)	
Sex			P < 0.001
Female	178216(99.1)	458(96.0)	
Male	1560(0.9)	19(4.0)	
Race			0.564
white	141247(78.6)	379(79.5)	
Black	20387(111.3)	47(9.9)	
Other	18142(10.1)	51(10.7)	
Grade			P < 0.001
Well	35041(19.5)	32(6.7)	
Moderately	72244(40.2)	136(28.5)	
Poorly	63637(35.4)	262(54.9)	
Undifferentiated	727(0.4)	3(0.6)	
Unknown	8127(4.5)	44(9.2)	
Stage			P < 0.001
I	91242(50.8)	201(42.1)	
II	55848(31.1)	130(27.3)	
III	18512(10.3)	1116(24.3)	
IV	8719(4.8)	25(5.2)	
Unknown	5455(3.0)	5(1.0)	
Tumor size			P < 0.001
T0	180(0.1)	12(2.5)	
T1	106807(59.4)	245(51.4)	
T2	51113(28.4)	116(24.3)	
T3	8636(4.8)	31(6.5)	
T4	7195(4.0)	63(13.2)	
NA	5845(3.3)	10(2.1)	
Node stage			P < 0.001
N0	120154(66.8)	266(55.8)	
N1	41370(23.0)	119(24.9)	
N2	9306(5.2)	54(11.3)	
N3	5462(3.0)	36(7.5)	
NX	3484(1.9)	2(0.4)	
Distant metastasis			0.358
M0	168093(93.5)	448(93.9)	
M1	8719(4.8)	25(5.2)	
Bone	5519(3.1)	8(1.7)	
Brain	623(0.3)	1(0.2)	
Liver	2302(1.3)	9(1.9)	
Lung	2828(1.6)	13(2.7)	
Unknown	2964(1.6)	4(0.8)	
Laterality			0.792
Left	91012(50.6)	250(52.4)	
Right	88552(49.3)	227(47.6)	
Paired	131(0.1)	0(0.0)	
Bilateral	34(0)	0(0.0)	
Unknown	47(0)	0(0.0)	
ER			P < 0.001
Negative	33827(18.8)	173(36.3)	
Positive	140777(78.3)	271(56.8)	
Borderline	115 (0.1)	2(0.4)	
Unknown	5057(2.8)	31(6.5)	
PR			P < 0.001
Negative	52290(29.1)	241(50.5)	
Positive	121560(67.6)	201(42.1)	
Borderline	277(0.2)	2(0.4)	
Unknown	5649(3.1)	33(6.9)	
HER2			P < 0.001
Negative	138627(77.1)	168(35.2)	
Positive	27962(15.6)	248(52.0)	
Borderline	4190(2.3)	9(1.9)	
Unknown	8997(5.0)	52(10.9)	
Subtype			P < 0.001
HR+/Her2−	116697(64.9)	139(19.1)	
HR+/Her2+	19188(10.7)	129(27.0)	
HR−/Her2+	8703(4.8)	115(24.1)	
TN	21670(12.1)	28(5.9)	
Unknown	13518(7.5)	66(13.8)	
Radiotherapy			P < 0.001
No	88867(49.4)	336(70.4)	
Yes	89570(49.8)	140(29.4)	
Unknown	1339(0.7)	1(0.2)	
Surgery			P < 0.001
Mastectomy	970786(50.5)	63(83.0)	
BCS	72845(40.5)	396(13.2)	
Other	16145(9.0)	18(3.8)	
LN surgery			P < 0.001
SLNB	124024(62.9)	257(53.9)	
ALND	50216(37.1)	215(45.1)	
Unknown	5536(3.1)	5(1.0)	
Status			0.006
Alive	166796(92.8)	427(89.5)	
Dead	12980(4.3)	50(10.5)	
Breast cancer	7797(4.5)	34(7.4)	
Other	5183(0.3)	16(3.5)	

*P values calculated by Pearson Chi squared testing; Bold if statistically significant, P < 0.05. PD: paget’s disease, IDC: invasive ductal carcinoma, y: years, BCS: breast-conserving surgery, HR: hormone receptor, TN: triple negative, LN: lymph node, SLNB: sentinel lymph node biopsy, ALND: axillary lymph node dissection.

**Table 2:** Cox proportional hazards regression model analysis of overall survival and breast cancer-specific mortality in patients with PD-IDC.

Variables	OS		BCSM	
	aHR (95% CI)	P-value	aHR (95% CI)	P-value
Age at diagnosis, y				
<35	Reference		Reference	
35–49	2.217(0.189, 26.056)	0.527	0.62(0.053, 7.261)	0.04
50–64	1.527(0.145, 16.047)	0.725	0.525(0.031, 5.49)	0.602
≥65	9.464(0.901, 99.457)	0.061	1.128(0.096, 13.693)	0.924
Sex	0.935(0.60, 2.34)	0.179	1.019(0.112, 3.636)	0.257
Race				
white	Reference		Reference	
Black	1.253(0.361, 4.351)	0.723	1.222(0.143, 4.307)	0.084
Grade				
Well	Reference		Reference	
Moderately	0.853(0.087, 8.392)	0.892	220.2(0.0, 2.65E24)	0.835
Poorly	1.825(0.198, 16.833)	0.595	508.2(0.0, 6.08E24)	0.81
Undifferentiated	2.818(0.117, 67.64)	0.523	5090.7(0.0, 6.52E24)	0.742
Stage				
I	Reference		Reference	
II	3.867(0.708, 21.372)	0.118	0.752(0.05, 10.313)	0.831
III	4.33(0.544, 34.221)	0.166	7.703(0.547, 73.108)	0.096
IV	28.68(2.60, 316.29)	0.006	23.97(1.972, 291.19)	0.013
Tumor size				
T0	Reference		Reference	
T1	1972.8(0.0, 3.84E28)	0.798	1744.2(0.0, 1.24E39)	0.859
T2	1361.6(0.0, 2.69E28)	0.808	6428.7(0.0, 4.73E39)	0.835
T3	466.9(0.0, 9.28E28)	0.836	7134.5(0.0, 8.4E38)	0.867
T4	2131.4(0.0, 4.23E28)	0.796	7593.5(0.0, 5.54E39)	0.832
Node stage				
N0	Reference		Reference	
N1	0.893(0.245, 3.245)	0.863	1.515(0.402, 1.637)	0.216
N2	1.761(0.282, 11.0)	0.545	1.817(0.609, 2.063)	0.364
N3	2.133(0.374, 12.178)	0.394	2.565(0.297, 2.898)	0.193
Distant metastasis				
M0	Reference		Reference	
M1	6.302(0.38, 104.521)	0.199	2.46(0.98, 61.898)	0.584
Laterality				
Left	Reference		Reference	
Right	1.215(0.56, 2.638)	0.623	1.139(1.062, 1.221)	0.435
ER				
Negative	Reference		Reference	
Positive	0.839(0.096, 7.386)	0.874	0.324(0.579, 3.217)	0.336
PR				
Negative	Reference		Reference	
Positive	0.414(0.139, 1.234)	0.113	0.553(0.151, 2.019)	0.37
HER2				
Negative	Reference		Reference	
Positive	2.096(0.647, 6.785)	0.217	5.169(1.245, 21.456)	0.024
Subtype				
HR+/Her2-	Reference		Reference	
HR+/Her2+	3711.4(0.0, 2.43E10)	0.305	11148.1(0.0, 2.96E11)	0.285
HR-/Her2+	1081.3(0.0, 8.06E9)	0.387	559.9(0.0, 1.73E10)	0.473
TN	1576.7(0.0, 1.21E10)	0.363	589.9(0.0, 1.85E10)	0.469
Radiotherapy				
No	Reference		Reference	
Yes	1.098(0.472, 2.554)	0.827	0.976(0.541, 4.596)	0.404
Surgery				
BCS	Reference		Reference	
Mastectomy	0.345(0.106, 1.124)	0.078	0.262(0.052, 1.311)	0.103
LN surgery				
ALND	Reference		Reference	
SLNB	3.317(1.136, 9.687)	0.028	3.397(0.952, 12.11)	0.059

PD: paget’s disease, IDC: invasive ductal carcinoma, y: years, BCS: breast-conserving surgery, HR: hormone receptor, TN: triple negative, LN: lymph node, SLNB: sentinel lymph node biopsy, ALND: axillary lymph node dissection. aHR: adjusted hazard ratio (adjusted for age at diagnosis, sex, race, grade, stage, tumor stage, node stage, distant metastasis, laterality, ER, PR, Her2, subtype, radiotherapy, surgery and LN surgery), CI: confidence interval.
