# Supplementary material for: Surgical treatment in Paget’s disease with invasive ductal carcinoma: an observational study based on SEER
Source: Sci Rep. 2017 Apr 19;7:45510. doi: 10.1038/srep45510 (PMC5395813; doi:10.1038/srep45510)
Supplement: Supplementary Information [file srep45510-s1.pdf]

Surgical treatment in Paget's disease with invasive ductal carcinoma: an observational study based on SEER

Qi Wu[1*], Juanjuan Li[1*], Si Sun[2], Shan Zhu[1], Juan Wu[3], Qian Liu[1], Shengrong Sun[1]

[1]Department of Breast and Thyroid Surgery, Renmin Hospital of Wuhan University, Wuhan, Hubei, P. R. China;

[2]Department of Clinical Laboratory, Renmin Hospital of Wuhan University, Wuhan, Hubei, P. R. China;

[3]Department of Pathology, Renmin Hospital of Wuhan University, Wuhan, Hubei, P. R. China;

Supplementary 1. Patient characteristics between PD-IDC group and random cohorts with IDC

**Variables**	**PD-IDC**	**IDC**		
	**N= 477(%)**	**Cohort 1**	**Cohort 2**	**Cohort 3**
		**N= 509(%)**	**N= 490(%)**	**N= 555(%)**
**Survival (months)**	22.21±13.62	21.55±13.35	21.53±13.33	21.02±13.26
**Age at diagnosis, y**		0.892	0.769	0.223
**＜35**	15(3.1)	13(2.6)	12(2.4)	8(1.4)
**35-49**	100(21.0)	112(22.0)	108(22.0)	122(22.0)
**50-64**	164(34.4)	180(35.4)	178(36.3)	207(37.3)
**≥65**	198(41.5)	204(40.1)	192(39.2)	218(39.3)
**Sex**		**0.011**	**0.014**	**< 0.001**
**Female**	458(96.0)	502(98.6)	483(98.6)	552(99.5)
**Male**	19(4.0)	7(1.4)	7(1.4)	3(0.5)
**Race**		0.619	0.518	0.861
**white**	379(79.5)	413(81.1)	401(81.8)	434(78.2)
**Black**	47(9.9)	51(10.0)	47(9.6)	60(10.8)
**Other**	51(10.7)	45(8.8)	42(8.6)	61(11.0)
**Grade**		**< 0.001**	**< 0.001**	**< 0.001**
**Well**	32(6.7)	115(22.6)	113(23.1)	117(21.1)
**Moderately**	136(28.5)	205(40.3)	193(39.4)	223(40.2)
**Poorly**	262(54.9)	166(32.6)	161(32.9)	189(34.1)
**Undifferentiated**	3(0.6)	0(0.0)	0(0.0)	0(0.0)
**Unknown**	44(9.2)	23(4.5)	23(4.7)	26(4.7)
**Stage**		**< 0.001**	**< 0.001**	**< 0.001**
**I**	201(42.1)	275(54.0)	268(54.7)	263(47.4)
**II**	130(27.3)	148(29.1)	140(28.6)	177(31.9)
**III**	1116(24.3)	59(11.6)	55(11.2)	71(12.8)
**IV**	25(5.2)	17(3.3)	17(3.5)	26(4.7)
**Unknown**	5(1.0)	10(2.0)	10(2.0)	18(3.2)
**Tumor size**		**< 0.001**	**< 0.001**	**< 0.001**
**T0**	12(2.5)	0(0)	0(0)	0(0)
**T1**	245(51.4)	327(64.2)	319(65.1)	310(55.9)
**T2**	116(24.3)	130(25.5)	121(24.7)	167(30.1)
**T3**	31(6.5)	24(4.7)	23(4.7)	30(5.4)
**T4**	63(13.2)	14(2.8)	13(2.7)	31(5.6)
**NA**	10(2.1)	14(2.8)	14(2.9)	17(3.1)
**Node stage**		**< 0.001**	**< 0.001**	**< 0.001**
**N0**	266(55.8)	341(67.0)	330(67.3)	358(64.5)

**N1**	119(24.9)	114(22.4)	110(22.4)	124(22.3)
**N2**	54(11.3)	27(5.3)	24(4.9)	36(6.5)
**N3**	36(7.5)	19(3.7)	18(3.7)	23(4.1)
**NX**	2(0.4)	8(1.6)	8(1.6)	14(2.5)
**Distant metastasis**		0.327	0.385	0.744
**M0**	448(93.9)	487(95.7)	468(95.5)	522(94.1)
**M1**	25(5.2)	17(3.3)	17(3.5)	26(4.7)
**Bone**	8(1.7)	10(2.0)	19(2.0)	18(3.2)
**Brain**	1(0.2)	2(0.4)	2(0.4)	1(0.2)
**Liver**	9(1.9)	3(0.6)	3(0.6)	7(1.3)
**Lung**	13(2.7)	4(0.8)	4(0.8)	9(1.6)
**Unknown**	4(0.8)	5(1.0)	4(0.8)	7(1.3)
**Laterality**		0.794	0.84	
**Left**	250(52.4)	271(53.2)	260(53.1)	283(51.0)
**Right**	227(47.6)	238(46.8)	230(46.9)	272(59.0)
**ER**		< **0.001**	< **0.001**	< **0.001**
**Negative**	173(36.3)	67(13.2)	65(13.3)	104(18.7)
**Positive**	271(56.8)	420(82.5)	404(82.4)	435(78.4)
**Borderline**	2(0.4)	1(0.2)	1(0.2)	0(0.4)
**Unknown**	31(6.5)	21(4.1)	20(4.1)	16(2.9)
**PR**		< **0.001**	< **0.001**	< **0.001**
**Negative**	241(50.5)	117(23.0)	113(23.1)	158(28.5)
**Positive**	201(42.1)	366(71.9)	352(71.8)	381(68.6)
**Borderline**	2(0.4)	1(0.2)	1(0.2)	0(0.0)
**Unknown**	33(6.9)	25(4.9)	24(4.9)	16(2.9)
**Her2**		< **0.001**	< **0.001**	< **0.001**
**Negative**	168(35.2)	401(78.8)	386(78.8)	438(78.9)
**Positive**	248(52.0)	64(12.6)	61(12.4)	84(15.1)
**Borderline**	9(1.9)	11(2.2)	11(2.2)	10(1.8)
**Unknown**	52(10.9)	33(6.5)	32(6.5)	23(4.1)
**Subtype**		< **0.001**	< **0.001**	< **0.001**
**HR+/Her2-**	139(19.1)	343(67.4)	331(67.6)	349(62.9)
**HR+/Her2+**	129(27.0)	59(11.6)	55(11.2)	74(13.3)
**HR-/Her2+**	115(24.1)	12(2.4)	12(2.4)	30(5.4)
**TN**	28(5.9)	50(9.8)	48(9.8)	69(12.4)
**Unknown**	66(13.8)	45(8.8)	44(9.0)	33(5.9)
**Radiotherapy**		< **0.001**	< **0.001**	< **0.001**
**No**	336(70.4)	258(50.7)	249(50.8)	264(47.6)

**Yes**	140(29.4)	244(47.9)	235(48.0)	289(52.0)
**Unknown**	1(0.2)	7(1.4)	6(1.2)	1(0.4)
**Surgery**		**< 0.001**	**< 0.001**	**< 0.001**
**Mastectomy**	63(83.0)	266(52.3)	253(51.6)	273(49.2)
**BCS**	396(13.2)	201(39.5)	197(40.2)	236(42.5)
**Other**	18(3.8)	42(8.3)	40(8.2)	46(8.3)
**LN surgery**		**< 0.001**	**< 0.001**	**< 0.001**
**SLNB**	257(53.9)	355(69.7)	344(70.2)	384(69.2)
**ALND**	215(45.1)	139(27.3)	132(26.9)	151(27.2)
**Unknown**	5(1.0)	15(2.9)	14(2.9)	20(3.6)
**Status**		**0.044**	**0.02**	**< 0.001**
**Alive**	427(89.5)	474(93.1)	459(93.7)	513(92.4)
**Dead**	50(10.5)	35(6.9)	31(6.3)	42(7.6)
**Breast cancer**	34(7.4)	23(4.5)	19(3.9)	27(4.9)
**Other**	16(3.5)	12(2.4)	12(2.4)	15(2.7)

** P values calculated by Pearson Chi squared testing; Bold if statistically significant, P< 0.05*

The random cohorts with IDC are generated from entirety.

PD: paget's disease, IDC: invasive ductal carcinoma, y: years, BCS: breast-conserving surgery, HR: hormone receptor, TN: triple negative, LN: lymph node, SLNB: sentinel lymph node biopsy, ALND: axillary lymph node dissection.

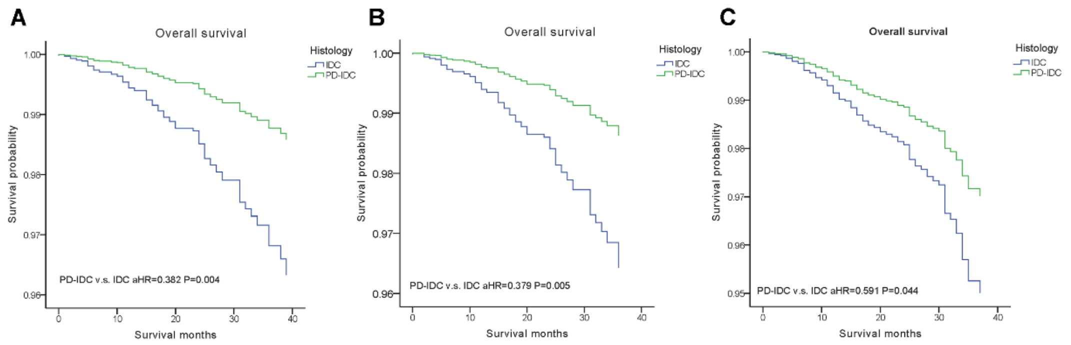

Supplementary 2. Weighted Kaplan-Meier curves of overall survival (OS) between PD-IDC group and random cohorts with IDC. (A). OS is analyzed between PD-IDC group and cohort 1. (B). OS is analyzed between PD-IDC group and cohort 2. (C). OS is analyzed between PD-IDC group and cohort 3. aHR: adjusted hazard ratio (adjusted for age at diagnosis, sex, race, grade, histology, stage, tumor stage, node stage, distant metastasis, laterality, ER, PR, Her2, subtype, radiotherapy, surgery and LN surgery).
